# Mediterranean Basin *Erica* Species: Traditional Uses, Phytochemistry and Pharmacological Properties

**DOI:** 10.3390/molecules30122616

**Published:** 2025-06-17

**Authors:** Khadijah A. Jabal, Maria Pigott, Helen Sheridan, John J. Walsh

**Affiliations:** 1 NatPro Centre, School of Pharmacy and Pharmaceutical Sciences, Trinity College Dublin, D02 PN40 Dublin, Ireland; jabalk@tcd.ie (K.A.J.); mpigott@tcd.ie (M.P.); hsheridn@tcd.ie (H.S.); 2 Department of Pharmacognosy, Faculty of Pharmacy, Umm Al-Qura University, Makkah 21955, Saudi Arabia

**Keywords:** mediterranean *Ericas*, diuretics, anti-microbials, anti-inflammatory, analgesics, anti-urolithiatics, essential oils, triterpenoids, polyphenolics, anthocyanidins

## Abstract

*Erica* species native to the Mediterranean basin are the principal *Ericas* that have found use in traditional medicine. Examples include treatments for urinary tract disorders, inflammatory conditions, gastrointestinal ailments and weight loss. This review critically evaluates the ethnobotanical usage, phytochemical profiles and pharmacological potential of the Mediterranean *Erica* species, including *Erica arborea* L., *Erica multiflora* L. and *Erica manipuliflora* Salisb. A wide spectrum of bioactive secondary metabolites has been identified across these species, notably pentacyclic triterpenes (e.g., lupeol, ursolic acid and oleanolic acid) and polyphenolics (e.g., myricetin and quercetin glycosides). Extracts of these species have demonstrated antioxidant, anti-inflammatory, analgesic, antimicrobial and diuretic activities in vitro and/or in vivo, providing pharmacological support for traditional uses. Phytochemical profiles vary by species, plant part, geography and extraction technique. Filsuvez^®^, comprising pentacyclic triterpenes from birch bark, has clinical approval for the treatment of partial thickness wounds associated with dystrophic and junctional epidermolysis bullosa. The undoubted reservoir of pentacyclic triterpenes and flavonoid glycosides in Mediterranean *Erica* species warrants further comprehensive mechanistic studies, toxicological evaluations and clinical validation.

## 1. Introduction

The Ericaceae family comprises 4250 species and 124 genera which include *Erica* (Heath), *Arbutus*, *Azalea*, *Vaccinium*, *Rhododendron* and *Calluna* [1,2,3,4,5]. The *Erica* genus encompasses a diverse range of evergreen shrubs recognized for their striking floral displays and remarkable adaptations to nutrient-poor soils. This genus consists of over 800 species distributed across various global regions, including South America, Europe, and the easternmost areas of Asia and South Africa, where the highest concentration of species can be found in the Cape Floristic Region [6]. Furthermore, *Erica* species are also present in other areas of Africa, particularly in the northern deserts situated between the equator and the Mediterranean Sea [7]. In general, *Erica* is one of the three most widely distributed genera of the Ericaceae within the Mediterranean region [8,9]. The name *Erica* comes from the ancient Greek word Ereiko, which means to break, referring to a tea made from a heath species that was believed to dissolve or break gallstones. The Swedish botanist Linnaeus used this term to define the genus in the eighteenth century [10,11]. The primary objective of this review is to present a thorough analysis of the traditional uses, phytochemistry and pharmacology of *Erica* species found in countries surrounding the Mediterranean basin namely *Erica arborea* L., *Erica multiflora* L., *Erica manipuliflora* Salisb., *Erica scoparia* L., *Erica australis* L., *Erica sicula* subsp. *sicula*, *Erica sicula* subsp. *bocquetii* (Peşmen) E.C.Nelson, *Erica spiculifolia* Salisb., *Erica terminalis* Salisb., *Erica lusitanica* Rudolphi, *Erica andevalensis* Cabezudo & J.Rivera, *Erica umbellata* L. and *Erica erigena* R.Ross. 

In southern European countries such as Italy, Portugal, Spain, France, Malta and Greece, as well as North African nations like Morocco, Algeria and Tunisia, and eastern Mediterranean countries including Turkey, Syria and Lebanon, specific *Erica* spp. are recognized for their applications in traditional medicine. They have been employed by local communities to address a variety of health conditions, including uses for their reputed anti-inflammatory, anti-urolithiatic, antioxidant, antibacterial, antiviral, antiseptic, astringent, antiulcer, analgesic and antihyperlipidemic effects [12,13]. Significant secondary metabolites of pharmacological interest isolated from these plants encompass polyphenolics, [9,14,15,16,17,18,19] triterpenes [12,20], anthocyanidins [21], essential oils [22,23] and fatty acids [24]. Notably, polyphenolics and triterpenoids are regarded as the key contributors to the therapeutic effects observed for various biological activities [12,16,25].

## 2. Characteristic Features and Geographical Distribution of *Erica* spp. in the Mediterranean Basin Region

Most *Erica* species are evergreen shrubs that attain heights ranging from 20 to 150 cm and possess needle-like leaves with some species growing several meters in height. Their adaptability allows them to thrive in a diverse array of soil types, including those that are nutrient-poor and characterized by low rainfall. A comprehensive description of each *Erica* is detailed in the textbook entitled *Hardy Heathers from the Northern Hemisphere* by E. Charles Nelson and summarized in Table 1 [7].

## 3. Traditional Uses of Erica Species

**Figure 1:**
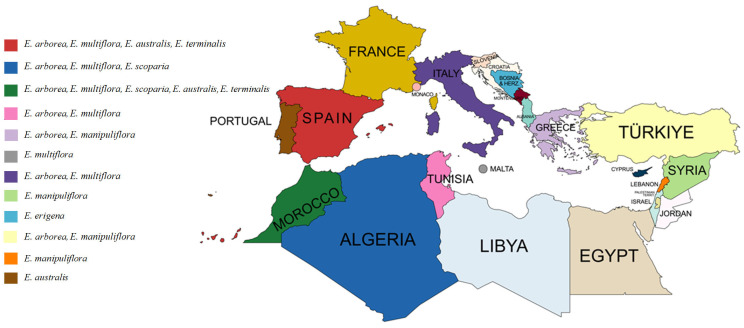
Countries of the Mediterranean basin and the *Erica* species with traditional use reports in those countries.

Ethnobotany explores the relationship between humans and plants, particularly the traditional uses of plants for medicine, food and other purposes [26,27,28]. It has been instrumental in discovering and developing many medicines from plant sources and preserving ethnobotanical knowledge is crucial to safeguard the socio-cultural heritage and practices of indigenous and local communities [29,30,31]. The Mediterranean basin, with approximately 25,000 plant species, is ethnobotanically rich [32,33,34,35] and ethnomedical uses of *Erica* species are reported throughout the region in Asian, African and European cultures (Figure 1). Reports on the ethnobotanical applications of *Erica* species from the literature are summarized in Table 2.

In Turkey, the *Erica* species *E. arborea* and *E. manipuliflora* Salisb. are widely used in traditional medicine for the treatment of a wide range of conditions such as urinary tract infections, kidney stones, hypertension and inflammatory diseases as well as for promoting weight loss [15,26,27,36,37,38,39,40,41,42,43,44,45,46,47,48,49,50,51,52,53,54,55,56,57,58,59,60,61,62]. There are also reports of the use of *E. manipuliflora* for skin conditions such as boils [62] and eczema [63] for gastrointestinal conditions such as constipation [64,65] and as an anthelmintic [66]. In Lebanon, Syria and Cyprus, similar traditional applications are reported for *E. manipuliflora* Salisb. [67,68,69,70]. In Algeria, *E. arborea* is used in traditional medicine for gastrointestinal illnesses including pinworm infection and stomachache and as a diuretic, anti-inflammatory and antimicrobial agent for a wide variety of conditions [8,71,72,73,74,75,76,77,78,79,80]. It also has reported use for nervousness [81]. *E. multiflora* preparations are employed in folk medicine in Tunisia [51,82,83] while Morocco is rich in *Erica* species and traditional medicinal applications in the region are reported for *E. multiflora*, *E. scoparia*, *E. terminalis*, *E. australis* and *E. arborea* [82,84,85,86,87,88,89,90,91,92]. In Southern European countries, the most extensively reported traditional medicinal uses of *Erica* species are in the treatment of urinary, prostate and kidney disorders with herbal infusions and decoctions employed for diuretic, anti-inflammatory and antiseptic purposes. In Spain, *E. multiflora* has reported use for wound healing [93] and *E. terminalis* for urinary tract infection [94], while *E. scoparia* was employed for its antiemetic and antispasmodic action [95]. In Portugal, *E. australis* has reported use for prostate and kidney health [96] while in Greece, *E. manipuliflora* Salisb. is reported as a treatment for prostate and urinary tract disorders [97]. *E. arborea* was employed in Greece for several conditions including rheumatism, anaemia and cystitis [97] while in Italy, it has reported use for nervous system disorders [98], oral infections [99], prostatic cystitis [100] and as a sedative in veterinary medicine [101]. *E. multiflora* was also valued for its sedative properties in Italy [102] and for its diuretic and antirheumatic effects and has reported use for urinary tract disorders in Malta [103]. In Bosnia and Herzegovina, *E. erigena* has been utilized for renal disorders [104].

Pharmacological effects of *Erica* preparations have been harnessed in regions of the Mediterranean basin since ancient times. Reference to *Erica* can be found in the writings of Dioscorides who described that cataplasms prepared from the leaves of *Erica* ‘do heal the biting of serpents’ [105]. Despite their extensive traditional applications, ethnopharmacological studies remain limited. Further toxicological, pharmacological and clinical research is necessary to validate these uses and refine medicinal formulations.

## 4. Chemical Constituents of Erica Species of the Mediterranean Basin

A diverse range of natural products have been identified in the Mediterranean *Erica* species. These include simple long-chain alkanes, alcohols, aldehydes and fatty acids/esters to several classes of terpenoids, phenolics, phenolic acids, flavonoids and flavonoid glycosides. In many cases the exact saccharide unit(s) attached to the phenols or flavonoids in glycosidic form have not been fully characterized and these are generally referred to as pentosides or hexosides.

### 4.1. Essential Oil Constituents

**Figure 2:**
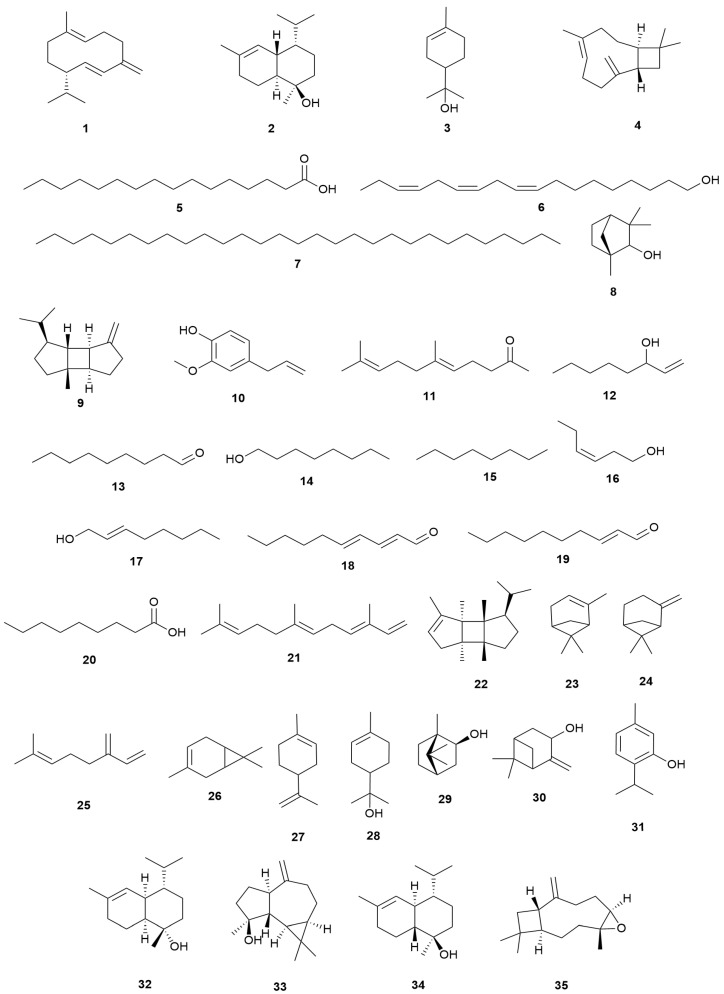
Structures of essential oil constituents profiled in Mediterranean *Erica* species.

The contents of mono- and sesquiterpenoids in the aerial parts, flowers and leaves of *E. manipuliflora* have been profiled with germacrene D (14.76%, 15.55% and 13.58%, respectively), τ-cadinol (7.53%, 4.11% and 8.96%), caryophyllene oxide (3.92%, 5.17% and 8.55%), β-caryophyllene (7.24%, 5.97% and 7.73%) and α-terpineol (6.85%, 6.14% and 4.18%) representing the dominant terpenoids present [106]. Sesquiterpene hydrocarbons (37.01%) were found to be dominant in the leaves while monoterpenoids (42.58%) predominated in the flowers [106]. Studies on the constituents of the essential oil of *E. arborea* leaves identified 75 components of which palmitic acid (33.3%), (Z,Z,Z)-9,12,15-octadecatrien-1-ol (6.6%) and nonacosane (6.1%) were the main constituents [22]. Terpenoids, including β-fenchyl alcohol, β-caryophyllene, β-bourbonene, ionol, *cis*-geranylacetone and germacrene-D represented the minor constituents together with eugenol [22]. A study on the constituents of *E. australis* essential oil, a plant with light pink, medium pink or dark pink flowers, was conducted following hydro-distillation of the dried flowering tops to investigate if flower color correlated with differences in volatile composition. No correlation was observed but 43 volatile constituents were identified. The most abundant compound was 1-octen-3-ol (33–38%), followed by *n*-nonanal (8–11%), *n*-octanol (6–7%), *n*-heptanol (4%), *cis*-3-hexen-1-ol (2–5%), 2-octen-1-ol (2–3%), 2-*trans*, 4-*trans*-decadienal (2–4%), 2-*trans*-decenal (2%) and nonanoic acid (2%) [107]. Only minor amounts of terpene constituents were present, namely geranyl acetone (1.7%), *trans*, *trans*-α-farnesene (0.8%) and a trace amount of *cis*-bourbonene [107]. Volatile terpenes are emitted by *Erica* spp. A study on *E. multiflora* in Spain found the principal monoterpenes emitted were α-pinene, β-pinene, β-myrcene, A^3^-carene and limonene, emissions varying seasonally and in response to experimental drought [108]. The composition of aerial parts of *E. spiculifolia* Salisb. essential oil following hydro-distillation has been comprehensively reported identifying 38 monoterpenes (46.2%), 30 sesquiterpenes (31.7%) and 2 diterpenes (0.4%) [109]. An additional 30 compounds, representing 14.3% of the oil comprised non-terpenoid constituents. The monoterpenes, α-terpineol (7.5%), endo-borneol (7.2%), pinocarveol (5.9%) and thymol (3.7%), were identified as the major oxygenated compounds. Within the sesquiterpene class, caryophyllene oxide (5.0%), caryophyllene (4.2%), τ-murrolol (3.5%), spathulenol (2.9%) and α-cadinol (2.3%) were profiled [109] (Table 3), (Figure 2).

### 4.2. Triterpenoids

**Figure 3:**
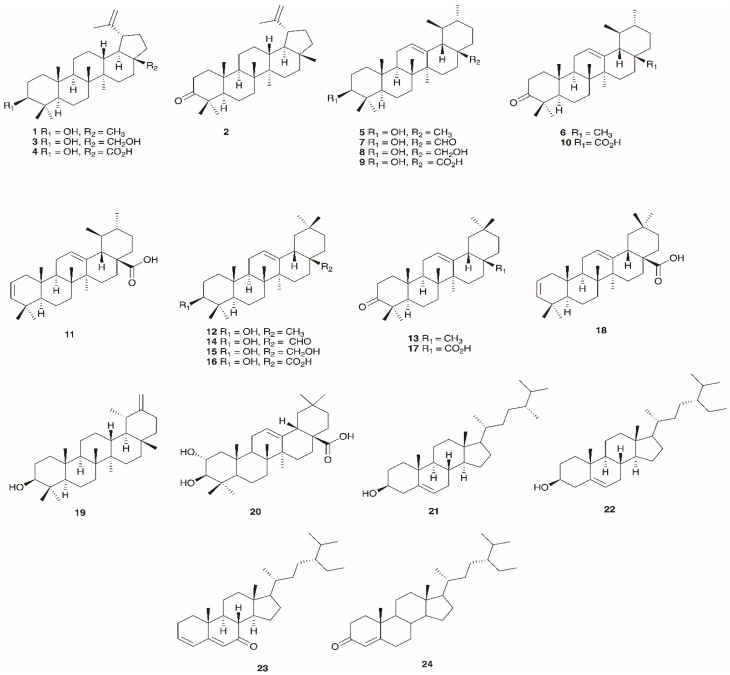
Structures of triterpenoid constituents profiled in Mediterranean *Erica* species.

Mediterranean heath species, and heaths generally, are a rich source of triterpenes with the pentacyclic triterpenes by far the most dominant class, especially those based on the ursane, oleanane and lupane scaffolds together with modest amounts of sterols and steroidal ketones. In-depth qualitative and quantitative analysis of the content of these constituents has been carried out on *E. arborea* by GC-MS [12] and to a lesser extent on *E. manipuliflora* [110], *E. andevalensis* [111] and *E. multiflora* [112]. In *E. arborea*, ursolic acid (14,889.49 μg/g) was a dominant triterpenoid in the profile followed by oleanolic acid (6022.89 μg/g), and while not separated by GC-MS, a mixture of lupeol/α-amyrin totaled 23,809.86 μg/g suggesting that these neutral triterpenoids may in fact be present in higher amounts [12]. A modest level of β-amyrin (2396.95 μg/g) was also present. The most dominant sterols were sitosterol and campesterol, 846.15 μg/g and 304.60 μg/g, respectively. Sitostenone and tremulone, 50.49 μg/g and 82.73 μg/g, respectively, were identified as the principal steroid ketones [12]. Ursolic acid has also been isolated from the aerial parts of *E. manipuliflora* [110] and *E. andevalensis* while α-amyrin has also been documented from *E. andevalensis* [111] and lupenone identified by HPLC in the leaves of *E. multiflora* [112] (Table 4), (Figure 3).

### 4.3. Phenolic Acids and Esters

**Figure 4:**
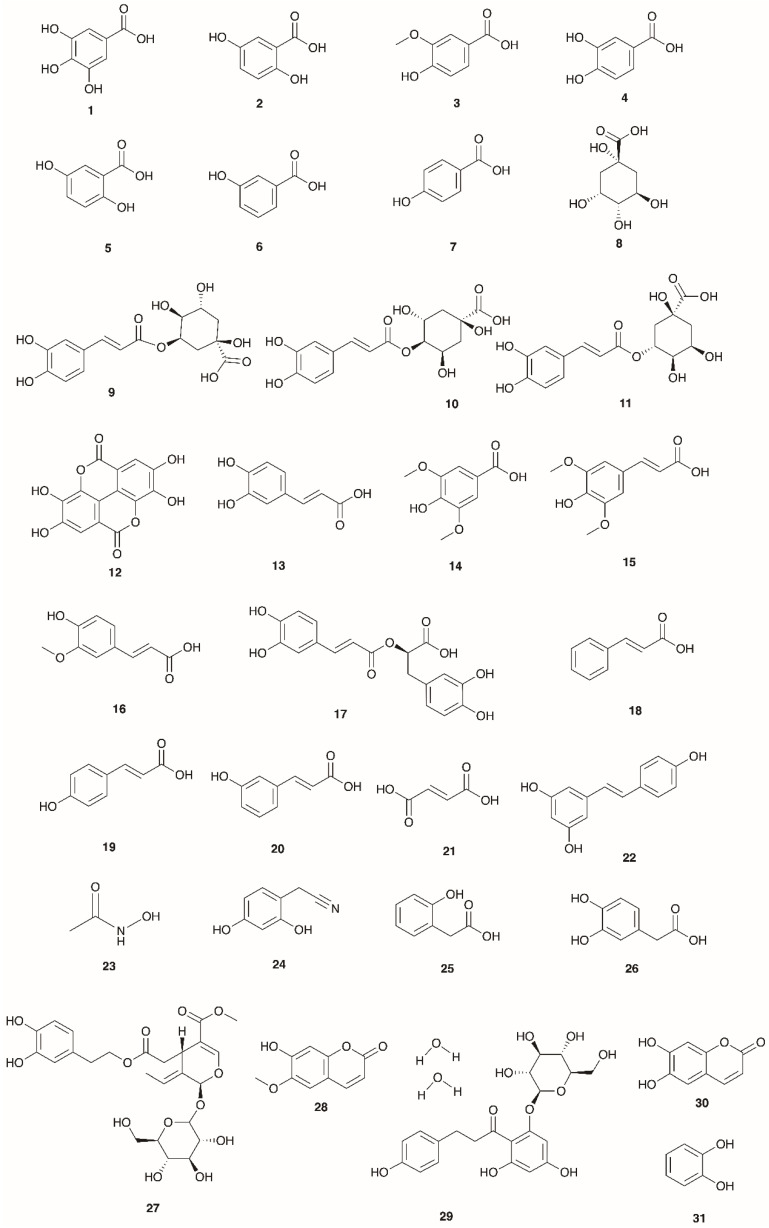
Structures of phenolic acids/esters/glycosides profiled in Mediterranean *Erica* species.

Many of the biosynthetic precursor compounds to flavonoids have also been identified in the Mediterranean *Erica* spp., including quinic, shikimic, gallic and phenyl acetic acids as well as the aryl C3 acids: cinnamic, coumaric, caffeic, ferulic and sinapic acids and esters/ether conjugate forms thereof [9,16,17,18,19,85,87,113,114,115,116,117]. Invariably, many of these constituents are present in lower amounts relative to the more extended flavonoid series except for 5-*O*-caffeoylquinic acid (583.28 mg/kg) in *E. arborea* [9]. Interestingly, in *E. multiflora* leaves, the level of 5-*O*-caffeoylquinic acid at 53.93 mg/kg [87] is 10-fold less than in *E. arborea*. Table 5, Figure 4 documents the species name, plant part from which the compound has been isolated, and the identification method used as well as the Mediterranean country of origin.

### 4.4. Phenylpropanoid Glucosides

**Figure 5:**
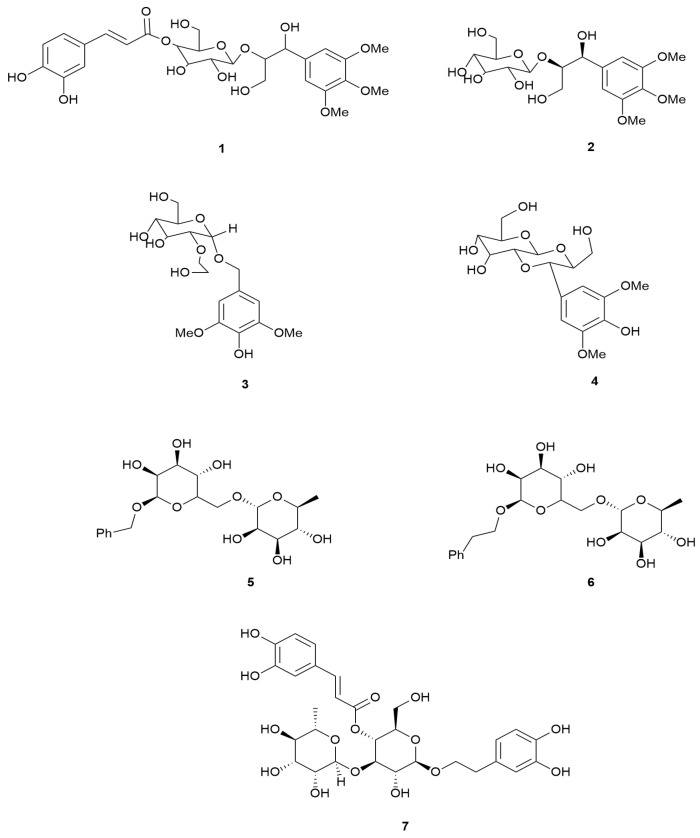
Structures of phenylpropanoid glucosides identified in *E. arborea*.

In Table 6, Figure 5, the phenylpropanoid glucoside series identified in *E. arborea* is documented where the aglycone moiety is linked via an ether to the sugar moiety; if there are two aglycones, an ester linkage may also be employed [119].

### 4.5. Flavonoids and Flavonoid Glycosides

**Figure 6:**
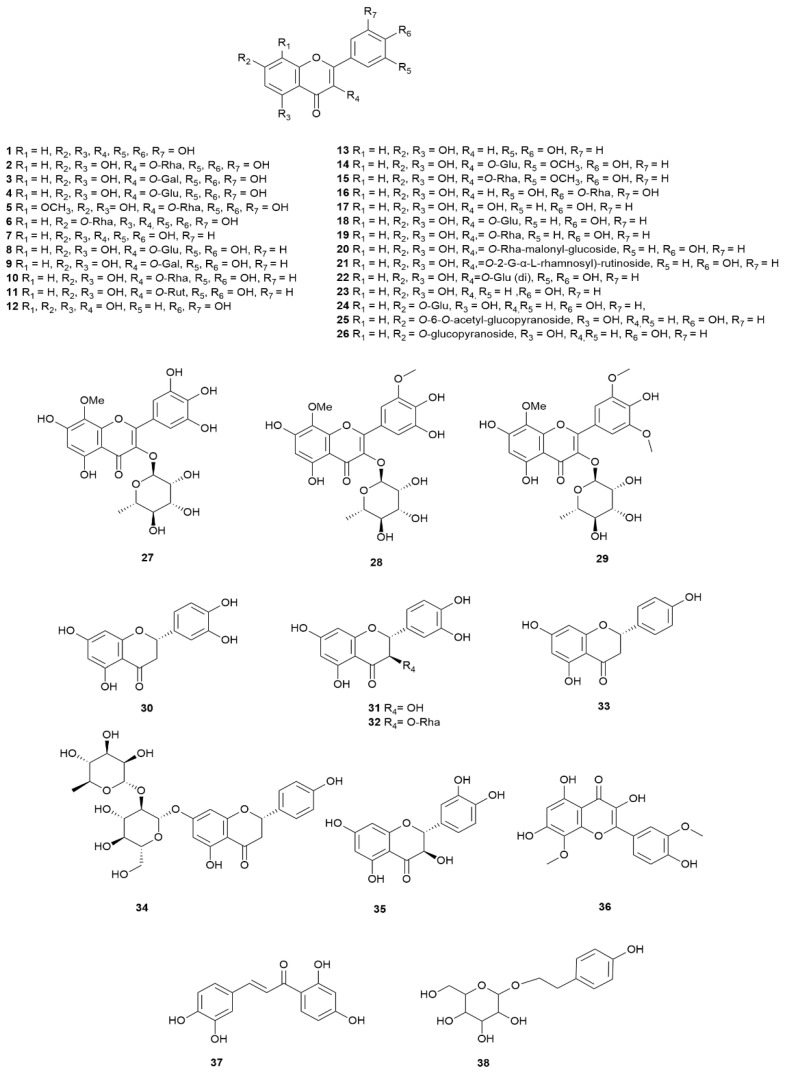
Structures of flavonoids and their glycosides profiled in Mediterranean *Erica* species.

Across all Mediterranean heath species, the most widely studied class of secondary metabolites are the flavonoids in both aglycone and glycoside forms. In many cases the exact mono/disaccharide has been identified, but the literature is full of examples where the sugar moiety has not been identified and the constituents are ambiguously specified as pentosides or hexosides, thus preventing true correlation of the active principle(s) with biological data. Of the flavonoid forms present, myricetin, quercetin, kaempferol and apigenin are the most common with some species also containing isorhamnetin and naringenin [9,16,18,19,85,87,113,115,117,120]. Both qualitative and quantitative analyses of the flavonoids in *E. arborea*, *E. scoparia*, *E. multiflora*, *E. australis* and *E. manipuliflora* have been documented [9,16,18,19,85,87,113,115,117,120]. While over 70 phenolic type compounds in *E. arborea* have been identified ranging from phenolic acids/esters to flavonoids in both free and glycoside form, the principal flavonoids identified were quercetin (598.72 mg/kg), quercetin 3-*O*-glucoside (633.41 mg/kg), kaempferol 3-*O*-glucoside (475.95 mg/kg), epicatechin (588.00 mg/kg) and catechin (27.43 mg/kg) when an accelerated solvent extraction procedure was performed on its dried powdered leaves [9]. A limited number of other flavonoids in free form have been identified and quantified including taxifolin, eriodictyol, luteolin and kaempferol [9]. Interestingly, the content of these constituents and that of the related glycoside forms varied considerably depending on the extraction method used ranging from microwave-assisted, ultrasound-assisted, and solvent-based to Soxhlet extraction methods. Of these, the ultrasound-assisted method proved to be the least efficient with the optimal method being accelerated solvent extraction [9]. LC-MS/MS analysis of a methanol extract of *E. multiflora* leaves harvested in Tunisia found that quercetin-3-*O*-glucoside and kaempferol-3-*O*-glucoside in almost equal proportions collectively constituted 60%, by area percentage, of the polyphenols present in the extract [117]. Methyl-dihydro-quercetin hexoside, myricetin and quercetin-3-*O*-rutinoside represented the other significant flavonoids present [117]. Overall, the total flavonoid content is significantly lower relative to *E. arborea* and *E. scoparia*. By far the most dominant flavonoid type in *E. scoparia* is myricetin which is present as myricetin-*O*-hexoside (184.38 mg/kg) and myricetin-*O*-rhamnoside (153.65 mg/kg) [87]. Several flavonoid glycosides have been identified, but not quantified, in *E. australis*. These include gossypetin glycoside, myricetin 3-*O*-glucoside, myricetin 3-*O*-rhamnoside, quercetin 3-*O*-rhamnoside, kaempferol 3-*O*-rhamnoside, quercetin acetyl-rhamnoside, quercetin 3-*O*-(6″-rhamnosyl) glucoside (rutin), quercetin 3-*O*-glucoside, isorhamnetin 3-*O*-glucoside, kaempferol 3-*O*-glucoside (astragalin) and quercetin 3-*O*-rhamnoside [18,115,121] (Table 7), (Figure 6).

### 4.6. Catechins

**Figure 7:**
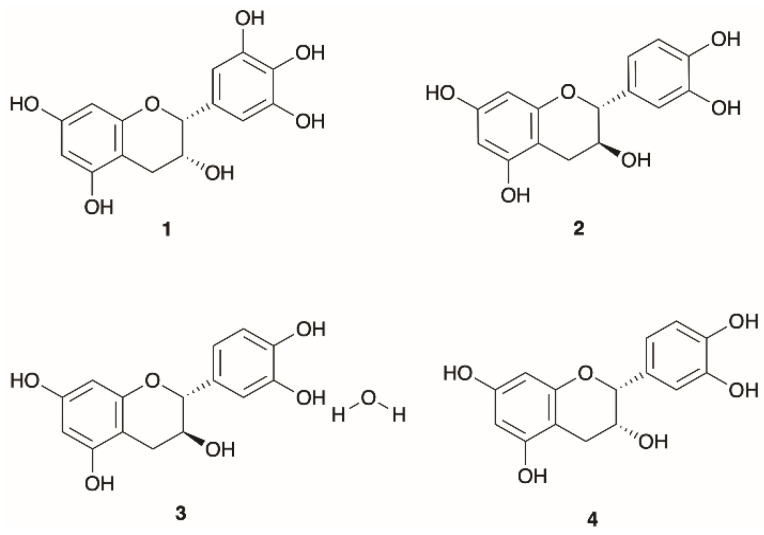
Structures of catechins profiled in Mediterranean *Erica* species.

A range of catechin compounds has been identified in Mediterranean *Ericas*, specifically in the species *E. australis*, *E. multiflora*, *E. andevalensis*, *E. manipuliflora*, and *E. arborea*. These compounds, which include epigallocatechin, catechin, catechin hydrate, and epicatechin are detailed in Table 8 and Figure 7 [9,18,19,24,74,85,113,116].

### 4.7. Anthocyanidins

**Figure 8:**
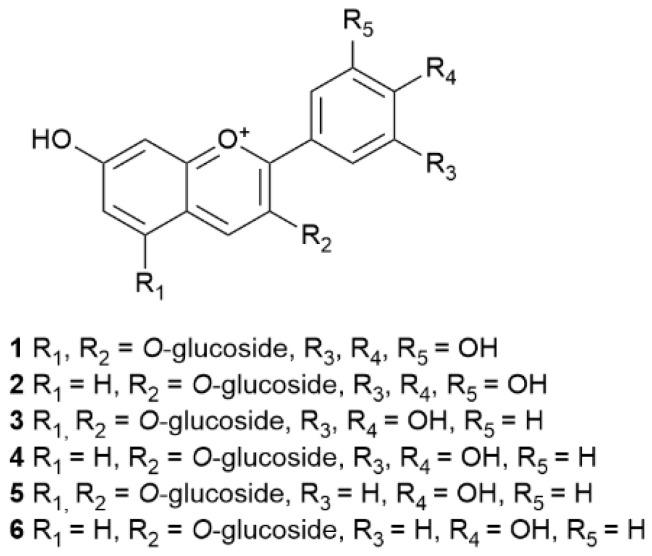
Structures of anthocyanidins profiled in *E. australis*.

Numerous anthocyanidins, including dimer and trimer compounds, have been identified in the Mediterranean *E. australis* such as delphinidin 3, 5-*O*-diglucoside, cyanidin 3,5-*O*-diglucoside, pelargonidin 3-5-*O*-diglucoside, delphinidin-3-*O*-glucoside, cyanidin-3-*O*-glucoside and pelargonidin-3-*O*-glucoside [113] (Table 9), (Figure 8).

A vast battery of secondary metabolites has been identified in Mediterranean *Erica* spp. These range from the terpenoid series (mono-, sesqui-, to the tri-terpenoids in particular) to polyphenolics where the flavonoid series predominates in both aglycone and glycoside presentations. Their characterization has relied significantly on the use of chromatographic methods, particularly GC-MS and HPLC with or without MS detection. Unambiguous characterization remains outstanding in some cases, particularly for flavonoid glycoside constituents. In this regard, further studies are warranted focusing on the use of NMR as a characterization tool.

## 5. Biological Activities

**Figure 9:**
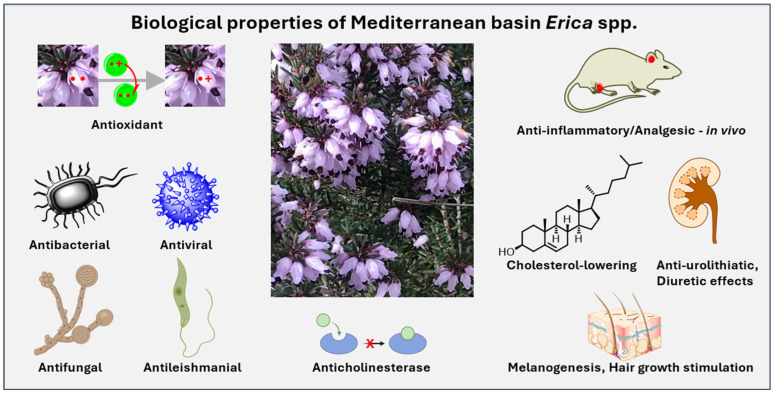
Biological properties of Mediterranean basin *Erica* species.

A vast array of biological activities has been documented for the Mediterranean basin Ericas. These are illustrated in Figure 9 and discussed in the next section.

### 5.1. Anti-Inflammatory Activity

Several studies have documented the anti-inflammatory activities of Mediterranean *Erica* spp. in vivo. Akkol et al. probed the anti-inflammatory activities of extracts of the aerial parts of *E. arborea*, *E. manipuliflora*, *E. bocquetii* and *E. sicula* subsp. *libanotica* collected in Turkey [50]. In this study, an aqueous extract and a methanol extract were examined for each species under investigation, as well as sequential solvent fractionations of the methanol extracts with chloroform, ethyl acetate and n-butanol. Of these extracts, the ethyl acetate extracts of *E. arborea*, *E. bocquetii* and *E. manipuliflora* at a dose of 100 mg/kg po inhibited the initial and second phases of the inflammatory response in a carrageenan-induced hind paw oedema model in mice with efficacy comparable to indomethacin at 10 mg/kg po. The same extracts also showed significant anti-inflammatory effects when used topically against ear oedema provoked by local application of 12-O-tetradecanoylphorbol-13-acetate (TPA) These extracts, as well as the ethyl acetate extract of *E. sicula* subsp. *libanotica*, also significantly inhibited inflammation in a prostaglandin E2 (PGE2)—induced hind paw oedema mouse model [51]. The traditional medicinal use of *Erica* spp. is often as an infusion or decoction in water. Akkol et al. did not observe significant anti-inflammatory effects in their in vivo models with oral administration of aqueous extracts of several *Erica* spp. at 100 mg/kg [50]. However, in a study on the anti-inflammatory effects of an aqueous extract of Algerian *E. arborea* aerial parts prepared by decoction, carrageenan-induced paw oedema and croton-oil-induced ear edema in mice were significantly reduced by the extract at doses of 250 and 500 mg/kg [8]. Amezouar et al. found that an ethanolic extract of Moroccan *E. arborea* leaves could inhibit carrageenan-induced paw oedema in the rat at 200 and 400 mg/kg po [125]. Amari et al. examined the topical and oral anti-inflammatory effects of hydro-methanolic extracts of *E. arborea* leaves and flowers. Both extracts showed significant anti-inflammatory activity in the xylene-induced ear oedema model, topical application of 0.5 mg/ear proving as effective as topical indomethacin at the same dose. In a parallel study, using croton oil to induce oedema, both extracts were again effective in reducing the swelling with the leaf extract proving marginally more potent. Both extracts were effective in these models when administered orally in the dose range of 100–500 mg/kg, and the effect was found to be dose-dependent [24].

### 5.2. Analgesic Activity

Studies on the analgesic activity of the Mediterranean *Erica* species have been documented. Using p-benzoquinone to induce abdominal constriction in mice, Akkol et al. showed that the ethyl acetate extracts of Turkish *E. arborea*, *E. manipuliflora* and *E. bocquetii* had notable antinociceptive activity at a dose of 100 mg/kg. These ethyl acetate extracts were prepared by sequential solvent fractionations of the methanol extracts with chloroform followed by ethyl acetate [50]. Nayebi et al. examined the analgesic effect of a hydromethanolic extract of the leaves and flowers Turkish *E. arborea* using the formalin test in mice as a model of tonic inflammatory pain. Intraperitoneal (i.p.) administration of the extract at a dose of 10 mg/kg decreased formalin-induced paw licking time in the early phase (0–5 min after formalin administration) and late phase (20–60 min after formalin administration). However, efficacy was not found to be dose-dependent. Higher doses of the extract at 20 mg/kg and 30 mg/kg did not produce significant reductions in paw licking time which the authors rationalized could be due to the presence of pro-algesic constituents in the plant extract [126].

### 5.3. Antioxidant Activity

Several studies have examined the antioxidant activity of *E. arborea*, *E. multiflora*, *E. scoparia* and *E. australis* using well-established antioxidant assays including the 2,2-diphenyl-1-picrylhydrazyl (DPPH), 2,2′-azino-bis(3-ethylbenzothiazoline-6-sulfonic acid (ABTS), CUPric reducing antioxidant capacity (CUPRAC) and ferric ion reducing antioxidant power (FRAP) assays. These assays typically measure the ability of compounds within an extract to donate an electron or hydrogen atom. Invariably associated with antioxidant studies are assays that determine the total phenol, flavonoid and tannin content. Of the *Erica* species, the most studied is *E. arborea*. In this context, Amari et al. conducted a series of sequential solvent extractions (using hexane, chloroform, ethyl acetate and water) on *E. arborea* sourced from Djebel of Tadergount mountain in Bejaia, Northern Algeria. In the DPPH assay, the flower extracts generally showed better activity than the leaf extracts, with IC_50_ values ranging from 38.18 to 60.16 μg/mL for leaves and 17.72 to 65.29 μg/mL for flowers. The ethyl acetate extract of the flowers was the most active, with the chloroform extract being the least effective. Of note, in the FRAP assay, the crude methanolic leaf extract was more effective than the flower extract with respective IC_50_ values of 2.91 and 6.22 μg/mL [127]. An ethanolic leaf extract of *E. arborea*, collected at an altitude of 1072 m in the Taza region of Morocco, displayed an IC_50_ of 10.22 μg/mL in the DPPH assay, which was comparable to butylhydroxytoluene 8.87 μg/mL. In the FRAP assay, the IC_50_ value obtained for the extract was 9.48 μg/mL [125]. In a more extensive study by Guendouze-Bouchefa et al. a defatted methanol extract of the aerial flowering parts of *E. arborea* demonstrated antioxidant activity against DPPH (IC_50_, 5.7 mg/L), ABTS (IC_50_, 6.8 mg/L) and superoxide anion radical with an antioxidant index value (AI_50_) of 213 mg/L. Using the same extraction methods and assays the respective IC_50_ values for the aerial flowering parts of *E. multiflora* were 10.2 mg/mL and 9.0 mg/mL with AI_50_ value of 261 mg/L in the superoxide anion radical assay [16]. While studies conducted using solvents of varying polarities will ultimately result in extracts with differences in phytochemical composition, the same can be anticipated if different extraction techniques are employed. This is exemplified by the work of Zengin et al., who used accelerated solvent extraction, microwave-assisted extraction, maceration, Soxhlet and ultrasound-assisted extraction methods to prepare extracts for investigation of the antioxidant activity of *E. arborea* leaf [9]. They found that the extract prepared by accelerated solvent extraction had significantly higher antioxidant activity when evaluated using the DPPH, ABTS, CUPRAC and FRAP assays than the extracts produced by the other extraction methods. A comparison of the antioxidant activity between *E. arborea* and *E. bocquetii* extracts prepared with a gradient polarity range of extraction solvents demonstrated that the alcoholic and aqueous extracts of *E. bocquetii* were more effective than the corresponding extracts for *E. arborea* [128]. At the level of the individual constituents, phenylpropanoid glucoside and flavonoid glycosides isolated from a methanol extract of *E. arborea* leaves showed antioxidant activity in the DPPH assay. The RC_50_ value for the phenylpropanoid glucoside, ericarborin, was 2.44 × 10^−5^ mg/mL vs. 2.88 × 10^−5^ mg/mL for quercetin [15]. In the same study, a series of flavonoid glycoside derivatives of dihydromyricetin, quercetin and apigenin were evaluated. Of these, quercetin 3-*O*-D-glucopyranoside was the most active, but still over forty-fold less active than quercetin. A comparison of the antioxidant activity of the hydroalcoholic extracts of the leaves and aerial parts of *E. multiflora* and *E. scoparia* was conducted using the DPPH and FRAP assays. In this study, the aerial extract of *E. scoparia* was the most effective with an IC_50_ value of 0.142 mg/mL vs. 0.611 mg/mL for *E. multiflora* in the DPPH assay. A similar correlation was observed in the FRAP assay, measured as ascorbic acid equivalents/mL, with an almost 3-fold difference in activity, 1.898 ASE/mL vs. 5.538 ASE/mL for *E. scoparia* over *E. multiflora*. The data can be rationalized based on the total phenolic content in their aerial parts, *E. scoparia*, calculated as 9528.93 mg/kg vs. 399.01 mg/kg for *E. multiflora* [87]. The aerial parts of *E. multiflora* were extracted separately with acetonitrile/water and water and evaluated in the DPPH assay. The water extract was more than two-fold more active in this assay with EC_50_ value of 8.55 μg/mL vs. 20.70 μg/mL for the acetonitrile/water extract [85]. A similar study using an aqueous extract of *E. australis* flowering parts found significant radical scavenging activity, with IC_50_ values of 6.7 µg/mL for the decoction and 10.5 µg/mL for the herbal infusion [121]. An ethanolic leaf extract of *E. multiflora* with was found to have an IC_50_ value of 10.85 mg/mL in DPPH and an EC_50_ value of 17.89 mg/mL in a ferric-reducing antioxidant assay [129]. A study conducted by Köroğlu et al. showed strong antioxidant activities for all extracts with different polarities of the aerial parts of *E. arborea*, in the following order: ethyl acetate > aqueous > crude > chloroform extract [13]. IC_50_ values against DPPH varied from 38.18 to 60.16 μg/mL for leaves and from 17.72 to 65.29 μg/mL for flowers [74]. In another study on the antioxidant activity of aqueous extracts of *E. australis* and *E. arborea* leaves and flowers, IC_50_ values ranged from 66.6 to 537.6 μg/mL in the DPPH assay and 296.3 to 4910.1 μg/mL in the ABTS assay, respectively, the aqueous extracts of leaves of *E. australis* and *E. arborea* possessing the highest antioxidant capacity and phenolic content [130]. Another study showed that the total phenolic content of an aqueous extract of *E. arborea* was 31.55 ± 0.45 mg GAE/g extract [17].

### 5.4. Antibacterial Activity

The discovery of new antimicrobial agents remains a key goal in drug development, particularly as antimicrobial resistance to our antibiotic armoury has emerged as one of the leading global threats to public health [131]. Microbial natural products have been the most prolific source of clinically used antimicrobial agents and it is anticipated that the natural world can continue to fuel the development pipeline [132]. Traditional medicinal knowledge can inform bioprospecting efforts, and in the case of the Mediterranean *Ericas*, traditional uses for wound healing and urinary tract infection have prompted antibacterial studies in these species. Guendouze-Bouchefa et al. evaluated the antibacterial effects of defatted methanol extracts of Algerian *E. arborea* and *E. multiflora* flowered aerial parts. The extracts were determined to have bactericidal activity against the Gram-positive strains tested but were inactive against the Gram-negative strains tested. The minimum inhibitory concentrations (MICs) of the *E. arborea* and *E. multiflora* extracts against *S. aureus* ATCC 6538 were 500 mg/L and 250 mg/L, respectively, while both extracts were determined to have a MIC against *S. aureus* C 100459 (MRSA) of 250 mg/L in broth microdilution assays, the authors considering plant extracts that display a MIC below 500 mg/L as active and worthy of further exploration. The extracts were inactive against *P. aeruginosa* AATCC 9027 and *E. coli* ATCC 25922. The effect of combining either plant extract with either cefotaxime or streptomycin was additive against *S. aureus* C100459 but the combinations had no beneficial interaction against *P. aeruginosa* [16]. Amari et al. also investigated the antibacterial activity of *E. arborea* harvested during flowering in north Algeria. Qualitative assessment by an agar disk diffusion test determined that a hydro-methanolic leaf extract and a hydro-methanolic flower extract inhibited the growth of three Gram-negative strains, namely *Escherichia coli* ATCC 11303, *Pseudomonas aeruginosa* ATCC 27853 and *Salmonella gallinarum* ATCC 700623, and three Gram-positive strains, namely *Bacillus cereus* ATCC 10987, *Micrococcus luteus* ATCC 27141 and *Staphylococcus aureus* ATCC 25923. MICs were subsequently determined. Relatively high concentrations of the extracts were needed to achieve inhibitory effects against all strains tested. Against *M. luteus*, the MICs were 1.60 mg/mL and 2.14 mg/mL for the flower extract and leaf extract, respectively, while against *P. aeruginosa* the leaf extract was slightly more effective with an MIC of 2.44 mg/mL in comparison to 9.13 mg/mL for the flower extract. Both extracts were determined as low mg/mL inhibitors of *B. cereus*, *S. aureus*, *E. coli* and *S. gallinarum*, with determined MICs in the range of 3.50–8.77 mg/mL [24]. In another study on *E. arborea* collected in Algeria, aqueous extracts of the leaves or flowers showed inhibitory potential in an agar diffusion assay against the Gram-positive bacteria, namely *Staphylococcus aureus* ATCC 25923, *Bacillus subtilus* CLAM20302 and *Bacillus cereus* CLAMH300, but were found inactive against the Gram-negative bacteria, namely *Escherichia coli* ATCC 25922, *Streptococcus* spp. and *Pseudomonas aeruginosa* ATCC 27853. The activities for both extracts were modest, but the leaf extract was found to be more potent, with reported MIC values in the range from 6.25 to 12.50 mg/mL in comparison to 25 mg/mL for the flower extract [80]. A study on the antimicrobial activities of the hexane, ethanol, methanol, ethyl acetate and aqueous extracts of the aerial parts of *E. arborea* L. and *E. bocquetii* P.F. Stevens from Turkey found that all the extracts of both species, ranging from non-polar to polar, had inhibitory activity against *Escherichia coli* ATCC 11230 G, and all extracts except the hexane extracts had inhibitory activity against *Escherichia coli* ATCC 29998 in a disk diffusion assay of 100 µg extract/disk. The ethyl acetate and aqueous extracts of *E. bocquetii* and the ethanol extract of *E. arborea* demonstrated activity against *Staphylococus aureus* ATCC 6538P while the ethyl acetate and ethanol extracts of *E. bocquetii* showed activity against *Salmonella typhimirium* CCM 5445. None of the extracts showed activity against *Staphylococcus epidermidis* ATCC 12228, *Enterobacter cloacae* ATCC 13047, *Enterococcus faecalis* ATCC 29212 and *Pseudomonas aeruginosa* ATCC 27853 [128]. A methanol extract, sequential to chloroform extraction, of the aerial parts of *E. multiflora* collected in Spain showed modest antimicrobial activity with a MIC of 1 g/L against *Staphylococcus aureus* ATCC 25923 and a MIC > 1 g/L against *Klebsiella pneumoniae* ATCC 18883 and *Mycobacterium phlei* CECT 3009. However, when analyzed by TLC bioautography, inhibition bands were not observed. While this may be due to limits of detection or limitations of the method, it is possible that the observed activity was due to additive effects or synergistic effects between multiple constituents of the extract [93]. Nefzi et al. found that the ethanol extract of *E. manipuliflora* leaves harvested in Tunisia had antibacterial activity against *Staphylococcus aureus* ATCC 29213 and *Escherichia coli* ATCC 8739 reporting an MIC against each strain of 0.04 mg/mL. The extract also demonstrated activity against *Salmonella typhimurium* NCTC 6017 and *Listeria monocytogenes* ATCC 7644, albeit less potent, with a MIC of 3.84 mg/mL in each case. These results did not indicate any selective antimicrobial activity based on differences in bacterial cell walls [129]. Modest antimicrobial activity has also been reported for an ethanol extract of flowering aerial parts of *E. manipuliflora* Salisb. collected in Turkey against *S. aureus* ATCC 25923, *E. coli* ATCC 25922 and *S. typhimurium* ATCC 14028 [19]. Tlas et al. examined the antibacterial activity of essential oils from *E. manipuliflora* Salisb. against two Gram-positive strains *Bacillus subtilis* and *Staphylococcus aureus* and two Gram-negative strains, *Escherichia coli* and *Salmonella enteritidis* by assessment of the minimum bactericidal concentration (MBC) of oil hydro-distilled from aerial parts collected before flowering and in full flowering in Syria. Both extracts achieved a full bactericidal effect on all strains tested. The authors reported greater sensitivity of Gram-positive strains to the extracts and that the essential oil of material collected during full flowering had greater potency against some of the tested strains. The MBCs against *Bacillus subtilis* for the oils from material collected before flowering and in full flowering were 16 mg/mL and 8 mg/mL, respectively, while the MBC against *Staphylococcus aureus* was 16 mg/mL for both extracts. The MBC of both extracts was 32 mg/mL against *Salmonella enteritidis* while the oil from the full flowering collection showed greater activity against *Escherichia coli* with a reported MBC of 16 mg/mL in comparison to 32 mg/mL for the oil from the before the flowering growth period [133].

While several studies report antibacterial effects for *Erica* species of the Mediterranean basin, the results are sometimes contradictory, a situation that is often encountered in antibacterial studies on natural products [134]. This is due to differences in methodologies, from extraction to strain selection to assay method, and also to a lack of consensus on what constitutes good activity, particularly in the context of a complex plant extract. Additionally, plant factors may contribute to biological effects such as plant part(s), season of harvesting and geographical location. In general, the antibacterial activities reported for the *Erica* species are attributed to the polyphenolic profiles of the plants, but little work has been carried out to fully delineate the constituent effects. Bio-guided fractionation and isolation can identify the contributing constituents and probe for additive or synergistic effects but dereplication methods are needed to avoid rediscovery of known, well-studied compounds. Overall, extracts of *Erica* species have been shown to have low to moderate antibacterial effects, particularly against Gram-positive strains. It is worth noting that low potency antimicrobials can still offer potential as part of combination therapies. Plant phenolics can act synergistically with antibiotics. Such synergies have therapeutic potential and are of particular interest in the restoration of activity of last-resort antibiotics against antimicrobial-resistant strains.

### 5.5. Antiviral Activity

Antiherpetic activity has been reported for *E. multiflora*. In a cytopathic effect (CPE) inhibition assay, a methanolic extract of the aerial parts of Tunisian *E. multiflora* showed high in vitro activity against Herpes simplex virus type 1 with an EC_50_ of 132.6 µg/mL in comparison to an EC_50_ of 0.8 µg/mL for the positive control, acyclovir. The extract showed complete cell protection against HSV-1-induced CPE at 500 µg/mL without toxicity to the host cells. In the same study, acetone and hexane extracts of the plant were found to be inactive [83].

### 5.6. Melanogenesis Stimulation

Upregulation of melanogenesis and tyrosinase activity are potential targets in the treatment of hypopigmentation disorders. An ethyl acetate leaf extract of *E. multiflora*, and one of its constituents, lupenone, were reported to stimulate melanogenesis in vitro by increasing the expression of tyrosinase enzyme. Lupenone treatment at 0.1 μM was comparable to treatment with 100 nM alpha-melanocyte stimulating hormone (α-MSH), a compound known to increase the melanin content of B16 cells [112].

### 5.7. Anti-Hyperlipidemia

Hyperlipidaemia represents a significant risk factor for the early development of atherosclerosis resulting in cardiovascular complications [135]. A plausible approach to target hyperlipidaemia is by diet and/or lipid lowering drugs [136]. In Eastern Morocco, *E. multiflora* is often used as an alternative therapy to treat hyperlipidemia. In this context, a study was conducted in a Triton-WR-1339-induced hyperlipidemic rat model to evaluate the anti-hyperlipidemic effects of an aqueous extract of *E. multiflora* flowers administered intragastically at a dose of 0.25 g/100 g BW in comparison with fenofibrate 65 mg/kg BW as the control lipid-lowering agent. The extract treatment significantly lowered total cholesterol and triglycerides at 7 h and 24 h after administration in comparison to the hyperlipidemic control group and to a greater extent than fenofibrate. The reduction in plasma total cholesterol by the extract was associated with a decrease in the LDL fraction, with HDL cholesterol not significantly altered by Triton WR-1339 induction or by the treatments [86]. Khlifi et al. determined the effects of a methanol leaf extract from *E. multiflora* harvested in Tunisia on mitigating the effects of metabolic syndrome in rats induced by a high-fat and high-fructose diet. The extract, at a dose of 250 mg/kg BW, prevented body weight gain, reduced total cholesterol, triglycerides and LDL-c and with an increase in HDL-c. Extract treatment also mitigated elevated glucose and insulin levels improving insulin homeostasis, reduced markers of inflammation and promoted antioxidant enzyme activities [117].

### 5.8. Acetylcholinesterase Inhibition

The naturally occurring acetylcholinesterase (AChE) inhibitor galantamine and rivastigmine, a semi-synthetic derivative of physostigmine, are used clinically for the treatment of early onset dementia of the Alzheimer’s type [137,138]. In addition, essential oils extracted from *Salvia officinalis* (Sage) and *Melaleuca alternifolia* (Tea tree) are noted AChE inhibitors [139,140]. In the context of the Mediterranean *Erica* spp. both a decoction (IC_50_, 257.9 µg/mL) and infusion preparation (IC_50_, 296.8 µg/mL) of the aerial parts of *E. australis* inhibited acetylcholinesterase [121]. A study evaluated *E*. *arborea* ethanol extracts prepared by different extraction techniques such as AChE and butyrylcholinesterase (BChE) inhibitors. The study compared ethanol extracts prepared by microwave-assisted, ultrasound-assisted, Soxhlet and accelerated solvent as well as by traditional solvent extraction. In general, activity against both enzymes were dependent on the extraction method used with accelerated solvent extraction proving optimal. The activity of the extracts against AChE and BChE were in the range of 3.71–4.91 mg galantamine equivalents (GALAE)/g and 5.52–6.18 mg GALAE/g, respectively [9].

### 5.9. Anti-Urolithiatic Activity

Urolithiasis is a kidney disorder in which stones form due to excessive mineral deposition in the urinary tract. It is a condition that affects 2–3% of the population. Approximately 80% of a kidney stone is composed of calcium oxalate mixed with calcium phosphate [141]. Two important processes for kidney stone formation/crystal build up in the urinary tract are calcium oxalate nucleation and crystal aggregation, both phenomena that are relatively easily measured in vitro. In this context, hydro-methanolic extracts of *E. arborea* L. leaf and flower at concentrations of 62.5, 125, and 500 µg/mL were evaluated in both assays. In the nucleation assay across all concentrations used for both extracts, inhibition ranged from ~88% to 98% with slightly better inhibition for the flower extract. In the aggregation assay, inhibition was generally lower across all concentrations used with the leaf extract (75.63%) exhibiting slightly better activity over the flower extract (72.87%) at 500 µg/mL. The ability of both extracts to inhibit nucleation and aggregation may relate to calcium binding to flavonoid constituents present in *E. arborea* [24].

### 5.10. Diuretic Effect

Medicines that reduce fluid buildup in the body are known as diuretics. The classical drug in this class is furosemide. In this context a comparative study was conducted comparing the effectiveness of aqueous extracts of *E. multiflora* flowers to furosemide using a rodent model [82]. At a dose of 0.250 g/kg, the extract significantly increased urinary output of water and electrolytes excretion within 1 h, 4 h and throughout the 24 h study period. The effect was thought to be unrelated to the K^+^ plant content. A higher dose of 0.50 g/kg of the extract was especially effective [82].

### 5.11. Antifungal Activity

*E. arborea* plant material from a local market in Turkey was extracted with 95% ethanol and was found to have antifungal activity against *Aspergillus niger* and *Candida albicans* (ATTC 60192) in a disk diffusion assay [142]. However, in another study the hexane, ethanol, methanol, ethyl acetate and aqueous extracts of the aerial parts of *E. arborea* from Turkey, as well as *E. bocquetii*, showed no activity against *Candida albicans* [128]. In another study, aqueous extracts of the leaves or flowers of *E. arborea* from Algeria were found inactive against *Aspergillus flavus* and *Aspergillus niger* [80].

### 5.12. Antileishmanial Activity

Leishmaniases are parasitic diseases caused by various species of protozoa of the genus *Leishmania* and transmitted by biting sandflies. Leishmaniasis is a disease that affects some of the world’s poorest people and is associated with malnutrition and weakened immunity, population displacement and poor living conditions. There is a need for effective and affordable treatments for this disease in addition to prevention and control strategies. The methanol extract of *E. arborea* flower from Algeria showed significant leishmanicidal activity and reliable selectivity indices. It was most effective against *L. major* with an IC_50_ against the promastigote form of 43.98 μg/mL but also demonstrated activity against *L. infantum* promastigotes (IC_50_ = 61.27 μg/mL) and so may contain promising antileishmanial phytochemical constituents [73].

### 5.13. Hair-Growth-Promoting Activity

*E. multiflora* has been identified as possessing hair-growth-promoting activity. A study on plant material collected in Tunisia and extracted with 70% ethanol found that the extract promoted the growth of human follicular dermal papilla cells (HFDPCs) in vitro by stimulating cell mitosis. The hair-growth-promoting effect of the extract was also demonstrated in a murine in vivo model following subcutaneous injection at test sites, thought by the authors to be due to indirect stimulation of the anagen or growth phase of the hair cycle from the telogen or resting phase [84].

## 6. Toxicity of Erica Species

Research conducted by Sadki et al. on *E. multiflora* demonstrates promising results, indicating that even at high dosages, the *E. multiflora* extract does not display significant signs of toxicity [82]. Furthermore, a study by Amroun et al. explored the safety and toxicity of an aqueous extract of *E. arborea* (EAAE) in rats, emphasizing both acute and sub-acute toxicity evaluations. In the acute toxicity phase, rats were administered a single dose of 2000 mg/kg or 5000 mg/kg of EAAE, alongside distilled water as a control. The results were encouraging, showing no signs of toxicity or mortality over a 14-day monitoring period for either dosage in both male and female rats, which underscores the extract’s relative safety. In the sub-acute toxicity assessment, rats received daily doses of EAAE (250, 500, and 1000 mg/kg) for 28 days. Notably, no mortality or toxic effects were observed, and there were no abnormal behaviours or morphological changes detected in either sex. These findings strongly suggest that EAAE extract may be safe for consumption at the tested levels. Nevertheless, it would be beneficial to conduct further research to deepen our understanding of its safety and potential effects [8].

## 7. Conclusions and Perspectives

The field of plant-based medicines continues to flourish but oftentimes the reputed traditional use of such products is not supported by validated studies at the phytochemical, pharmacological or clinical level. This situation is precisely the case with the Mediterranean heaths which have found widespread traditional use for the treatment of a myriad of conditions including inflammation, pain, diabetes, urinary tract infections, weight loss treatments and gallstones. Where pharmacological studies are reported on the Mediterranean *Ericas* these are oftentimes not supported by a complete phytochemical analysis of the extract used in the study. This is an important omission stemming from the multitude of factors that affect phytochemical content including genetics, climatic conditions, plant age, cultivation conditions, geographical location and microenvironments within the same geographical location. Additionally post-harvest treatment, the method of extraction and the extraction solvent are factors affecting the phytochemical composition of a final extract.

Studies have been reported on *E. arborea* regarding its triterpenoid, phenolic acid, flavan-3-ol, pro-anthocyanidin and flavonoid/glycoside constituents. However, in many cases the exact sugar unit or its point of attachment on the flavonoid backbone is not known, thus making a direct correlation between phytochemical constituents present and outcomes of pharmacological studies challenging. In this context, further spectroscopic studies are warranted using advanced nuclear magnetic resonance spectroscopy techniques combined with high-resolution mass spectroscopy and x-ray crystallography to unambiguously confirm the identity of the phytochemical constituents. Once the identity of the constituents is known in a given plant, detailed qualitative and quantitative studies should follow to precisely establish the levels of each constituent. In this regard, further studies can build upon the data generated to date on Mediterranean *Erica* spp. where GC/GCMS has been used to profile the volatile constituents and higher order terpenoid constituents following derivatization. While HPLC/LCMS has been used for qualitative and quantitative studies of what might loosely be termed the phenolic constituents, HPLC has also been utilized for the analysis of pentacyclic triterpenes at low wavelength detection, *circa* 210 nm. This is challenging as many of the long-chain hydrocarbon compounds present in *Erica* spp. also absorb at this wavelength.

In conclusion, a true correlation between traditional use and observed therapeutic effects is only valid if the plant material has been sourced from the precise region where it is used. In establishing a direct correlation, detailed phytochemical analysis of the plant material should be conducted in parallel with pharmacological studies. Nevertheless, Mediterranean *Ericas* have shown potential in a broad range of in vitro and/or in vivo assays that measure antioxidant, anti-inflammatory, analgesic and antimicrobial activity of extracts and individual constituents. Further studies to determine the quality, safety, and efficacy of Mediterranean *Ericas* in traditional medicine are warranted. Their richness in pentacyclic triterpenes, similar to those contained in the clinically approved birch bark extract, Filsuvez^®^, should serve as the impetus for future work with Mediterranean *Ericas* [143].

## Figures and Tables

**Table 1:** Morphological characteristics and geographical locations of *Erica* species in regions of the Mediterranean basin.

Species	Height	Leaf Morphology	Flower Morphology	Growing Regions in the Mediterranean Basin
*E. arborea* (Tree heath)	To 7 m	Leaves arranged in whorls of 3, linear, 5–7 mm in length	White or very pale pink, terminal on short leafy shoots in umbels of 2–4	Widely distributed in the region across southern Europe, northern Africa and to the east in countries including Turkey, Lebanon and Syria
*E. multiflora* (Many-flowered heath)	To 2.5 m	Thick, leathery leaves arranged in whorls of 3–5, linear, 10–15 mm in length and 1–1.5 mm broad	White to pink in axillary clusters of 1–4	Europe: eastern Spain and the Balearic Islands, southern France (including the northern tip of Corsica), Italy (including Lampedusa, Sardinia and Sicily), Malta and Gozo, southern coastal Croatia, Albania and north-west Greece. North Africa: Algeria, Morocco, Tunisia and Libya
*E. scoparia*, *E. scoparia* subsp. *scoparia* (Besom heath)	1 to 4 m	Leaves arranged in whorls of 3 or 4, linear, 4–10 mm in length	Inflorescences are numerous and crowded on shoots; individual inflorescence are umbels of 1–3 greenish flowers, rarely tinged with red, on very reduced lateral branchlets	Western Mediterranean basin. Europe: Portugal, Spain including the Balearic Islands, southern and south-western France including Corsica, north-western Italy and Sardinia. North Africa: Morocco, Algeria and Tunisia
*E. manipuliflora* (Whorled heath)	To 4 m	Leathery leaves arranged in whorls of 3 or 4, 3–9 mm in length	Inflorescences composed of several to many axillary umbels of 1–5 flowers on very short shoots, in varying shades of mauve, pink or rarely white	Italy, southern Croatia, Montenegro, Albania, Greece including Crete and the Ionian and Aegean islands, Turkey, Northern Cyprus, Syria and Lebanon
*E. australis* (Southern heath)	To 2.5 m	Leaves arranged in whorls of 4, linear in shape, to 7 mm in length	The inflorescences are terminal on leafy lateral shoots, flowers in 4 s, sometimes with subsidiary whorls, in varying shades of pale pink to lilac-link and sometimes white	Western Iberian Peninsula, in regions of Portugal and Spain, as well as in northern Morocco
*E. terminalis* (Corsican heath)	To 2–3 m	Leaves arranged in whorls of 4–5, lanceolate to linear, to 9 mm in length	Inflorescences are a single terminal umbel, or a compound inflorescence of several umbels on leafy lateral shoots, generally in pink to purple	Southwestern and southern Europe: Spain, Corsica and Italy including Sardinia. North Africa: Morocco
*E. sicula*. subsp. *sicula* (Sicilian heath)	To 0.6 m	Leaves arranged in whorls of 4 to 5, spreading or ascending, linear 3–13 mm in length	Inflorescences with 2–8 flowers in terminal umbels on main or axillary shoots in pale to deep pink, sometimes white	Italy (specifically Sicily), Libya, Turkey (specifically Anatolia), and areas of Cyprus, Lebanon and Libya
*E. sicula* subsp. *bocquetii* (Bocquet’s heath)	To 0.25 m often spreading to form hummocks	Leaves arranged in whorls of 3 to 4, spreading or ascending, linear, 3–6 mm in length	Flowers 2–3, rarely solitary, in umbel, terminal on main or axillary shoots in pale to deep pink	Western Asia: Turkey (Anatolia only) above 1000 m altitude
*E. spiculifolia* (Balkan heath, Spike heath)	To 15 cm	Arranged in irregular whorls of 2 to 6 or spirally arranged, linear-lanceolate, 4–6 mm in length, although the leaves found in inflorescences can be longer, reaching up to 9 mm	The inflorescences typically consist of a terminal raceme with 8–40 flowers, in bright pink to red-pink, very rarely white	South-eastern Europe: Bosnia and Herzegovina, Montenegro, Macedonia, Albania and Greece. Western Asia: northern Turkey
*E. umbellata* (Dwarf Spanish heath)	To 0.6 m	Leaves arranged in whorls of 3, linear, small at 2–5 mm length and 0.5 mm in width	Inflorescences are terminal umbels of 1–6 flowers, in pink to purple, occasionally white	Spain and Portugal and northern Morocco
*E. andevalensis*	To 2 m	Arranged in whorls of 4 to 5, with young shoot internodes ~1.5 mm long, while older shoot internodes range from 5 to 7.5 mm long, ovate, ~5 mm in length and to 2.5 mm in width	Inflorescences are terminal and umbellate in dark pink, rarely white	South-western Iberian Peninsula only, in regions of Spain and Portugal
*E. lusitanica* (Spanish heath, Portuguese heath)	To 4.5 m	Leaves arranged in whorls of 4 (sometimes in 3 s), linear with edges parallel or lanceolate and narrowing slightly to tip, 7 mm in length and 0.5 mm in width	Inflorescences are numerous and crowded towards ends of shoots, 1–4 flowers in each terminal umbel at tip of short, leafy lateral shoots, in white, often tinged pink in the bud	Iberian Peninsula: Small pockets widely scattered in southern and western Portugal and south-western Spain

**Table 2:** Summary of the traditional uses of Mediterranean *Erica* spp. in countries of the Mediterranean basin from the literature.

Plant Species (Local Name)	Region	Plant Part(s)	Preparation	Uses/Treatment	Reference(s)
**Western Asia**					
**Turkey**					
*E. manipuliflora* Salisb.	Turkey	Flowers, branches and leaves	Decoction/Infusion	Obesity	[27]
*E. manipuliflora* Salisb.(Püren)	Karaisalı	Branches and flowers	Infusion	Weight loss	[36]
*E. manipuliflora* Salisb. (Piren, Funda)	Marmaris, Muğla	Leaves	Infusion	Weight loss and as a diuretic	[37]
*E. manipuliflora* Salisb. (Püren and Funda)	Dalaman, Muğla	Leaves and flowers	Decoction	Weight loss and for diabetes treatment	[38]
*E. arborea* (Funda)	Mount Ida (Balıkesir)	Leaves	Infusion	Weight loss	[39]
*E. arborea* (Briar, Tree heath)	Turkey	Leaves and seeds	Infusion	For treatment of obesity	[27]
*E. arborea* (Püren, Piren)	Edremit Bay (Balıkesir)	Flowers and branches	Infusion	Asthma	[40]
*E. arborea* (Funda)	Gönen, Balıkesir	Flowering branches	Decoction	Diuretic	[41]
*E. arborea* (Funda, Piren, Süpürge otu, Süpürge çalısı)	Çatalca	Fruit	Externally	Foot wounds and mouth sores	[42,43,44]
*E. arborea* (Funda, Piren, Süpürge otu, Süpürge çalısı)	Çatalca	Fruit	Internally	Foot and mouth disease in animals	[42]
*E. arborea* (Çalısüpürgesi, pirançalısı)	Düzce province	Flowers	Infusion	Sooth itching in anal fissure	[45]
*E. arborea* (Süpürge)	South part of İzmit Gulf	Aerial parts	Decoction	Hypertension	[46]
*E. arborea* (Funda)	Kastamonu province	Leaves	Infusion	Inflammation, urinary tract infection and kidney stones	[47]
*E. arborea*	Turkey	Leaves and flowers	Not defined	Constipation, diuretic, hypertension, renal lithiasis, inflammation, sooth itching in anal fissure, urinary tract infection, kidney stones, renal fluid flow, poor eyesight, snakebites, stomach problems, sleeping disorders, mouth sores, poor circulation, colds, gout, lumbago, muscular aches, motion sickness, hangover cure	[15,48,49]
*E. arborea*	Turkey	Leaves	A glass of 5% decoction or infusion	Edema	[50]
*E. arborea* (Funda/Tree heath)	Sourced in Gaziantep herbal markets, Turkey	Leaves and shoots	Infusion	Urinary and respiratory disorders	[51]
*E. arborea*	Turkey	Flower tips	Decoction	Renal lithiasis, diuretic and a urinary antiseptic	[52]
*E. manipuliflora* Salisb. (Süpürge)	Western region of Turkey	Shoots	Infusion	Diuretic	[53]
*E. manipuliflora* Salisb. (Acram)	In the district of Antakya	Flowering parts	Not defined	Anthelmintic	[54,55]
*E. manipuliflora* Salisb. (Funda)	Kazdağı National Park, West Turkey	Leaves	Not defined	Urinary tract infection and appetite suppressant	[56]
*E. manipuliflora* Salisb. (Püren, Pürenotu, Süpürgeotu, Sükürteotu)	Turkey	Flowers and branches	Decoction Internal/drink one glass 3 times a day for 4–8 weeks	Kidney stones	[57]
*E. manipuliflora* Salisb. (Püren, Pürenotu, Süpürgeotu, Sükürteotu and Funda)	Alaşehir (Manisa)	Flowers, branches and leaves	Decoction (one glass 3 times daily) or infusion	Diabetes, hypertension, constipation, arthritis, obesity, nephralgia, gastrointestinal diseases, diuretic, ureter infection, sedative and kidney stones	[28,37,39,40,41,54,57,58,59,60,61,62]
*E. manipuliflora* Salisb. (Funda, Püren)	Turkey	Flowers and leaves	Decoction	Hypertension	[59,60]
*E. manipuliflora* Salisb. (Piren, Püren)	Datça Peninsula, South-west Turkey	Flowers	Infusion	Sedative	[61]
*E. manipuliflora* Salisb. (Funda, Süpürge out and Püren)	Turkey	Aerial parts	External as ointment with olive oil	Boils	[62]
*E. manipuliflora* Salisb. (Funda, Süpürge out, Püren)	Turkey	Fruit, flowers and branches	As ointment with olive oil	Eczema	[63]
*E. manipuliflora* Salisb. (Püren)	Ceylanlı village of Kırıkhan district of Hatay area	Stems	Not defined	Diuretic, constipation, arthritis and weight loss	[64]
*E. manipuliflora* Salisb.and *E. arborea*	Turkey	Aerial parts	Infusion	Constipation, urethritis and diuretic effects	[65]
*E. manipuliflora* Salisb. (Püren)	Antakya	Flowers	Infusion	Anthelmintic properties	[66]
**Lebanon**					
*E. manipuliflora* Salisb. (Khalanj laqui, Shantaf)	Lebanon	Flowers and twigs	Decoction	Rheumatism and antineuralgic	[67]
*E. manipuliflora* Salisb.	Lebanon	Flowers	Not defined	Sedative	[68]
**Syria**					
*E. manipuliflora* Salisb. (Ajram)	Western Region (Latakia and Tartus)	Flowers	Decoction	Sedative, diuretic, gout and urinary tract infection, while the heather honey of the plant is commonly used as a tonic, expectorant, to treat rheumatism asthma, dysmenorrhea and arthritis, as a laxative, disinfectant for the respiratory tract, urinary tract infections, acute nephritis, relieving nerve pain, depression, treating insomnia, bladder and prostate pain and enlargement	[69]
**Syria, Lebanon, Turkey, Cyprus**					
*E. manipuliflora* Salisb.	Syria, Lebanon, Turkey, Cyprus	Flowers, leaves, branches and shoots	Infusion/Decoction and boiled	Urethritis, arthritis, weight loss, diuretic, constipation	[70]
**North Africa**					
**Algeria**					
*E. arborea* (Khlenj)	Algeria	Aerial parts and stems	Oral, infusion or decoction	Diuretic, anti-inflammatory, astringent, antiulcer and antimicrobial agent, treat hypertension, kidney inflammations, urolithiasis, renal lithiasis, pinworm infection, urinary infections, stomachache and prostate diseases	[8,71,72,73,74,75]
*E. arborea* (Bouhadad, khlenj)	Tadergount, Derguina-Bejaia, North of Algeria	Flowers, leaves and aerial parts	External/Internal	Kidney stones, eczema, urinary and gastric diseases, inflammation, microbial infections and snakebites	[74]
*E. arborea* (Elkhlilanj)	Algeria	Aerial parts	Infusion/Decoction	Lithiasis and urinary infections	[75]
*E. arborea* (Akhlendj)	The Djurdjura National Park	Flowers	Infusion	Physical weakness and anxiety	[76]
*E. arborea* (Axlenǧ)	Kabylia region	Leaves/Roots	Decoction, Cataplasm	Rheumatism	[77]
*E. arborea* (Elkhlilanj)	The region of Chlef	Stems	Infusion	Gastrointestinal illnesses including pinworm infection and stomachache	[78]
*E. arborea* (Akheloundj)	Kabylia area (North Algeria)	Flowers	Internal	Urinary stone	[79]
*E. arborea* (Akheloundj)	Kabylia area (North Algeria)	Flowers	External	Freckles	[79]
*E. arborea*	The Setifian Tell, East Algeria	Flowers	Infusion	Acute and chronic urinary infection	[80]
*E. arborea* (Akhlenj)	Djurdjura Biosphere Reserve	Flowers	Decoction	Indigestion and nervousness	[81]
**Tunisia**					
*E. multiflora*	Kalaa Sghira	Aerial parts	Not defined	Diuretic, urinary infections, tranquilizing, astringent and prostate cancer	[51,82,83]
**Morocco**					
*E. multiflora*	Morocco	Not defined	Not defined	Diuretic	[82]
*E. multiflora* (Khlenj)	Morocco	Not defined	Not defined	Hypertension, inflammation, hyperlipidemia and atherosclerosis	[84,85,86]
*E. scoparia* and *E. multiflora*	Northern Morocco	Not defined	Infusion	Analgesic and anti-inflammatory activities	[87]
*E. multiflora*	Northern Morocco	Not defined	Infusion	Liver function repair effects and antilithiatic actions	[88]
*E. terminalis* Salisb. (El Khalanj)	Zemmour and Zayane	Whole plant	Decoction or oral	Veterinary use for lameness	[89]
*E. arborea* (Khlenj)	Bni-Leit and Al-Oued districts, a part of the Natural Regional Park of Bouhachem	Seeds	Decoction or local application	Headaches and sexual diseases	[90]
*E. australis*	Morocco	Not defined	Infusion	Diuretic, antiseptic and to treat infected wounds	[91]
**Southern European countries**					
**Spain**					
*E. multiflora* (Brezo o Erica)	Spain	Aerial parts	Not defined	Wound healing	[92,93]
*E. terminalis* Salisb.	Western part of Granada (southern Spain)	Flowers	Decoction	Urinary infections	[94]
*E. scoparia* (Bruc)	L’Alt Empordà and Les Guilleries, located in North East Catalonia	Floral tops	Infusion	Antiemetic and antispasmodic	[95]
**Portugal**					
*E. australis*	In Vilar de Perdizes	Flower	Not defined	Prostate, bladder and kidney disease	[96]
**Greece**					
*E. arborea*	Mt. Pelion	Leaves and stems	Decoction	Rheumatism, anemia, cystitis, diarrhea, diuretic and acne	[97]
*E. manipuliflora* Salisb. (Sousora)	Mt. Pelion	Leaves, flowers and stems	Decoction	Urinary tract diseases and treat prostate	[97]
**Italy**					
*E. arborea* (Ulece)	Peninsula Sorrentina, Campania, Southern Italy	Not defined	Not defined	Nervous system disorders in folk veterinary medicine	[98]
*E. arborea* (Urxa and Socche)	Eastern Riviera (Liguria)	Not defined	Not defined	Mouth infections	[99]
*E. arborea*	Roccamonfina region in Campania, Southern Italy	Flowers	Decoction	Prostatic cystitis	[100]
*E. arborea*	Inland Southern Italy	Stems	Not defined	Sedative in veterinary medicine	[101,102]
**Malta**					
*E. multiflora* (Xkattapietra)	Gozo, Malta	Aerial parts	Decoction	Urinary tract disorders	[103]
**Bosnia and Herzegovina**					
*E. erigena* R.Ross (Erika)	Middle, southern and western Bosnia and Herzegovina	Aerial parts	Not defined	Renal disorders	[104]

**Table 3:** Essential oil constituents identified in Mediterranean *Erica* species.

No.	Compound	Species	Location	Plant Part(s)	Identification	Reference
**1**	Germacrene-D	*E. arborea*	Algeria	Leaves	GC/MS	[22]
		*E. manipuliflora*	Turkey	Aerial parts	GC/MS	[106]
**2**	τ-Cadinol	*E. manipuliflora*	Turkey	Aerial parts	GC/MS	[106]
**3**	α-Terpineol					
**4**	β-Caryophyllene	*E. manipuliflora*	Turkey	Aerial parts	GC/MS	[106]
		*E. arborea*	Algeria	Leaves	GC/MS	[22]
**5**	Palmitic acid	*E. arborea*	Algeria	Leaves	GC/MS	[22]
**6**	(Z,Z,Z)-9,12,15-Octadecatrien-1-ol					
**7**	Nonacosane					
**8**	β-Fenchyl alcohol					
**9**	β-Bourbonene					
**10**	Eugenol					
**11**	Geranylacetone	*E. arborea*	Algeria	Leaves	GC/MS	[22]
		*E. australis*	Portugal	Flowering aerial parts	GC/MS	[107]
**12**	1-Octen-3-ol	*E. australis*	Portugal	Flowering aerial parts	GC/MS	[107]
**13**	*n*-Nonanal					
**14**	*n*-Octanol					
**15**	*n*-Heptanol					
**16**	*cis*-3-Hexen-1-ol					
**17**	2-Octen-1-ol					
**18**	2-*trans*, 4-*trans*-Decadienal					
**19**	2-*trans*-Decenal					
**20**	Nonanoic acid					
**21**	*trans*, *trans*-α-Farnesene					
**22**	*cis*-Bourbonene					
**23**	α-Pinene	*E. multiflora*	Spain	Foliar emissions	GC/MS	[108]
**24**	β-Pinene					
**25**	β-Myrcene					
**26**	A^3^-Carene					
**27**	Limonene					
**28**	α-Terpineol	*E. spiculifolia* Salisb.	Bulgaria	Aerial parts	GC/MS	[109]
**29**	endo-Borneol					
**30**	Pinocarveol					
**31**	Thymol					
**32**	τ-Murrolol					
**33**	Spathulenol					
**34**	α-Cadinol					
**35**	Caryophyllene oxide	*E. spiculifolia* Salisb.	Bulgaria	Aerial parts	GC/MS	[109]
		*E. manipuliflora*	Turkey	Aerial parts	GC/MS	[106]

**Table 4:** Triterpenoids identified in Mediterranean *Erica* species.

No.	Compound	Species	Location	Plant Part(s)	Identification	Reference
**1**	Lupeol	*E. arborea*	Algeria	Aerial parts	GC-MS	[12]
**2**	Lupenone	*E. arborea*	Algeria	Aerial parts	GC-MS	[12]
		*E. multiflora*	Tunisia	Leaves	HPLC	[112]
**3**	Betulin	*E. arborea*	Algeria	Aerial parts	GC-MS	[12]
**4**	Betulinic acid					
**5**	α-Amyrin	*E. arborea*	Algeria	Aerial parts	GC-MS	[12]
		*E. andevalensis*	Spain	Aerial parts	IR, MS, NMR	[111]
**6**	α-Amyrenone	*E. arborea*	Algeria	Aerial parts	GC-MS	[12]
**7**	Ursolic aldehyde					
**8**	Uvaol					
**9**	Ursolic acid	*E. arborea*	Algeria	Aerial part	GC-MS	[12]
		*E. manipuliflora*	Turkey	Aerial parts	NMR and MS	[110]
		*E. andevalensis*	Spain	Aerial parts	IR, MS, NMR	[111]
**10**	3-Oxoursolic acid	*E. arborea*	Algeria	Aerial parts	GC-MS	[12]
**11**	Ursa-2,12-dien-28-oic acid					
**12**	β-Amyrin					
**13**	β-Amyrenone					
**14**	Oleanolic aldehyde					
**15**	Erythrodiol					
**16**	Oleanolic acid					
**17**	3-Oxooleanolic acid					
**18**	Olean-2,12-dien-28-oic acid					
**19**	Taraxasterol					
**20**	Maslinic acid					
**21**	Campesterol					
**22**	Sitosterol					
**23**	Tremulone					
**24**	Sitostenone					

**Table 5:** Phenolic acids/esters/glycosides identified in Mediterranean *Erica* species.

No.	Compound	Species	Location	Plant Part(s)	Identification	Reference
**1**	Gallic acid	*E. arborea*	Turkey	Not defined	LC–ESI–MS/MS	[17]
		*E. manipuliflora*	Turkey	Aerial parts	LC-MS/MS	[19]
		*E. multiflora*	Tunisia	Aerial parts	HPLC	[85]
		*E. australis*	Portugal	Leaves and flowers	HPLC	[113]
**2**	Gentisic acid	*E. scoparia*	Spain	Leaves	TLC	[114]
		*E. australis*	Portugal	Leaves and flowers	HPLC	[113]
		*E. australis*	Spain	Flowers, stems and roots	TLC	[115]
**3**	Vanillic acid	*E. manipuliflora*	Turkey	Aerial parts	LC-MS/MS	[19]
		*E. arborea*	Turkey	Leaves	HPLC-LTQ OrbiTrap MS	[9]
		*E. multiflora*	Tunisia	Aerial parts	HPLC	[85]
		*E. australis*	Spain	Leaves, stems and roots	TLC	[115]
		*E. scoparia*	Spain	Leaves	TLC	[114]
		*E. andevalensis*	Spain	Leaves	HPLC	[18]
		*E. australis*	Spain	Leaves	HPLC	[18]
		*E. arborea*	Spain	Leaves	HPLC	[18]
**4**	3,4-Dihydroxybenzoic acid	*E. arborea*	Turkey	Not defined	LC–ESI–MS/MS	[17]
		*E. manipuliflora*	Turkey	Aerial parts	LC-MS/MS	[19]
		*E. scoparia*	Spain	Leaves	TLC	[114]
		*E. arborea*	Turkey	Leaves	HPLC-LTQ OrbiTrap MS	[9]
**5**	2,5-Dihydroxybenzoic acid	*E. arborea*	Turkey	Leaves	HPLC-LTQ OrbiTrap MS	[9]
		*E. arborea*	Turkey	Not defined	LC–ESI–MS/MS	[17]
**6**	3-Hydroxybenzoic acid	*E. arborea*	Turkey	Not defined	LC–ESI–MS/MS	[17]
**7**	4-Hydroxybenzoic acid	*E. arborea*	Turkey	Leaves	HPLC-LTQ OrbiTrap MS	[9]
		*E. arborea*	Turkey	Not defined	LC–ESI–MS/MS	[17]
		*E. manipuliflora*	Turkey	Aerial parts	LC-MS/MS	[19]
		*E. australis*	Spain	Leaves, stems, roots and flowers	TLC	[115]
**8**	Quinic acid	*E. multiflora*	Tunisia	Leaves	LC–MS/MS	[117]
**9**	5-*O*-Caffeoylquinic acid	*E. arborea*	Turkey	Leaves	HPLC-LTQ OrbiTrap MS	[9]
**10**	4-*O*-Caffeoylquinic acid	*E. multiflora*	Morocco	Aerial parts	LC–DAD/ESI–MS	[87]
**11**	3-*O*-Caffeoylquinic acid (Chlorogenic acid)	*E. multiflora*	Tunisia	Leaves	LC–MS/MS	[117]
		*E. arborea*	Turkey	Not defined	LC–ESI–MS/MS	[17]
		*E. arborea*	Turkey	Leaves	HPLC-LTQ OrbiTrap MS	[9]
		*E. australis*	Portugal	Leaves and flowers	HPLC	[113]
**12**	Ellagic acid	*E. andevalensis*	Spain	Leaves	HPLC	[18]
		*E. australis*	Spain	Leaves	HPLC	[18]
		*E. arborea*	Spain	Leaves	HPLC	[18]
**13**	Caffeic acid	*E. arborea*	Turkey	Leaves	HPLC-LTQ OrbiTrap MS	[9]
		*E. arborea*	Spain	Leaves	HPLC	[18]
		*E. multiflora*	Algeria	Flowered aerial parts	HPLC–DAD–ESI-MS	[16]
		*E. manipuliflora*	Turkey	Aerial parts	LC-MS/MS	[19]
		*E. scoparia*	Spain	Leaves	TLC	[114]
		*E. australis*	Portugal	Leaves and flowers	HPLC	[113]
		*E. australis*	Spain	Roots	TLC	[115]
		*E. andevalensis*	Spain	Leaves	HPLC	[18]
		*E. australis*	Spain	Leaves	HPLC	[18]
**14**	Syringic acid	*E. arborea*	Turkey	Not defined	LC–ESI–MS/MS	[17]
		*E. scoparia*	Spain	Leaves	TLC	[114]
**15**	Sinapic acid	*E. arborea*	Turkey	Not defined	LC–ESI–MS/MS	[17]
		*E. australis*	Portugal	Leaves and flowers	HPLC	[113]
		*E. australis*	Spain	Roots	TLC	[115]
**16**	Ferulic acid	*E. arborea*	Turkey	Not defined	LC–ESI–MS/MS	[17]
		*E. scoparia*	Spain	Leaves	TLC	[114]
		*E. australis*	Spain	Leaves, stems, roots and flowers	TLC	[115]
**17**	Rosmarinic acid	*E. arborea*	Turkey	Not defined	LC–ESI–MS/MS	[17]
**18**	Cinnamic acid	*E. australis*	Portugal	Leaves and flowers	HPLC	[113]
		*E. andevalensis*	Spain	Seeds	HPLC	[116]
		*E. andevalensis*	Spain	Leaves	HPLC	[18]
**19**	*p*-Coumaric acid	*E. arborea*	Turkey	Leaves	HPLC-LTQ OrbiTrap MS	[9]
		*E. multiflora*	Algeria	Flowered aerial parts	HPLC–DAD–ESI-MS	[16]
		*E. scoparia*	Spain	Leaves	TLC	[114]
		*E. australis*	Portugal	Leaves and flowers	HPLC	[113]
		*E. australis*	Spain	Leaves, flowers and roots	TLC	[115]
		*E. australis*	Spain	Leaves	HPLC	[18]
		*E. andevalensis*	Spain	Leaves	HPLC	[18]
		*E. andevalensis*	Spain	Seeds	HPLC	[116]
**20**	*m*-Coumaric acid	*E. australis*	Spain	Leaves	HPLC	[18]
		*E. arborea*	Spain	Leaves	HPLC	[18]
		*E. andevalensis*	Spain	Seeds	HPLC	[116]
**21**	Fumaric acid	*E. manipuliflora*	Turkey	Aerial parts	LC-MS/MS	[19]
**22**	Resveratrol	*E. manipuliflora*	Turkey	Aerial parts	LC-MS/MS	[19]
**23**	Acetohydroxamic acid	*E. manipuliflora*	Turkey	Aerial parts	LC-MS/MS	[19]
**24**	2,4-Dihydroxy-phenyl acetonitrile	*E. scoparia*	Spain	Leaves	NMR	[118]
**25**	2-Hydroxyphenyl acetic acid	*E. scoparia*	Spain	Leaves	NMR	[118]
**26**	3,4-Dihydroxyphenyl acetic acid	*E. arborea*	Turkey	Not defined	LC–ESI–MS/MS	[17]
**27**	Oleuropein	*E. manipuliflora*	Turkey	Aerial parts	LC-MS/MS	[19]
**28**	Scopoletin	*E. australis*	Spain	Leaves, flowers, stems and roots	TLC	[115]
**29**	Phloridzin dihydrate	*E. manipuliflora*	Turkey	Aerial parts	LC-MS/MS	[19]
**30**	Aesculetin	*E. australis*	Spain	Leaves, flowers, stems and roots	TLC	[115]
**31**	Pyrocatechol	*E. arborea*	Turkey	Not defined	LC–ESI–MS/MS	[17]

**Table 6:** Phenylpropanoid glucosides identified in *E. arborea*.

No.	Compound	Species	Location	Plant Part(s)	Identification	Reference
**1**	Ericarborin	*E. arborea*	Turkey	Leaves	NMR	[15]
**2**	1,2-Erythro-1-(3,4,5-trimethoxyphenyl)-2-(β-D-glucopyranosyloxy) propan-1,3-diol	*E. arborea*	Turkey	Leaves and Flowers	NMR and MS	[118]
**3**	Ericarboside					
**4**	Ficuscarpanoside B					
**5**	Benzylrutinoside					
**6**	Phenethylrutinoside					
**7**	Verbascoside	*E. arborea*	Turkey	Not defined	LC–ESI-MS/MS	[17]

**Table 7:** Flavonoids and their glycosides profiled in Mediterranean *Erica* species.

No.	Compound	Species	Location	Plant Part(s)	Identification	Reference(s)
**1**	Myricetin	*E. arborea*	Turkey	Leaves	HPLC-LTQ Orbitrap MS	[9]
		*E. manipuliflora*	Turkey	Aerial parts	LC-MS/MS	[19]
		*E. manipuliflora*	Greece	Aerial parts	NMR	[120]
		*E. andevalensis*	Spain	Leaves	HPLC	[18]
		*E. australis*	Spain	Leaves	HPLC	[18]
		*E. arborea*	Spain	Leaves	HPLC	[18]
		*E. multiflora*	Tunisia	Leaves	LC–MS/MS	[117]
		*E. australis*	Portugal	Leaves and flowers	HPLC	[113]
		*E. australis*	Spain	Flowers and roots	TLC	[115]
**2**	Myricetin 3-*O*-rhamnoside	*E. scoparia*	Morocco	Leaves	LC–DAD/ESI–MS	[87]
		*E. australis*	Portugal	Flowering aerial parts	HPLC-DAD and HPLC-ESI-MS	[121]
**3**	Myricetin 3-*O*-galactoside	*E. andevalensis*	Spain	Flowering tops	IR, MS, NMR	[122]
		*E. andevalensis*	Spain	Flowering tops	IR, MS, NMR	[123]
**4**	Myricetin 3-*O*-glucoside	*E. multiflora*	Tunisia	Leaves	LC–MS/MS	[117]
		*E. australis*	Portugal	Flowering aerial parts	HPLC-DAD and HPLC-ESI-MS	[121]
**5**	8-Methoxy-myricetin 3-*O*-rhamnoside	*E. arborea*	Turkey	Leaves	HPLC-LTQ OrbiTrap MS	[9]
		*E. scoparia*	Morocco	Aerial parts	LC–DAD/ESI–MS	[87]
**6**	Myricetin 7-*O*-rhamnoside	*E. arborea*	Turkey	Leaves	HPLC-LTQ Orbitrap MS	[9]
**7**	Quercetin	*E. australis*	Spain	Leaves, flowers and roots	TLC	[115]
		*E. arborea*	Turkey	Leaves	HPLC-LTQ Orbitrap MS	[9]
		*E. manipuliflora*	Turkey	Aerial parts	LC-MS/MS	[19]
		*E. multiflora*	Algeria	Flowered aerial parts	HPLC–DAD–ESI-MS	[16]
		*E. multiflora*	Morocco	Aerial parts and leaves	LC–DAD/ESI–MS	[87]
		*E. multiflora*	Tunisia	Aerial parts	HPLC	[85]
		*E. manipuliflora*	Greece	Aerial parts	NMR	[120]
		*E. australis*	Portugal	Leaves and flowers	HPLC	[113]
**8**	Quercetin 3-*O*-β-D-glucopyranoside	*E. arborea*	Turkey	Leaves	NMR	[15]
		*E. arborea*	Turkey	Leaves	HPLC-LTQ Orbitrap MS	[9]
		*E. multiflora*	Tunisia	Leaves	LC–MS/MS	[117]
**9**	Quercetin 3-*O*-galactoside (Hyperoside)	*E. arborea*	Turkey	Not defined	LC–ESI–MS/MS	[17]
**10**	Quercetin 3-*O*-α-L-rhamnopyranoside	*E. arborea*	Turkey	Leaves	NMR	[15]
		*E. arborea*	Turkey	Leaves	HPLC-LTQ Orbitrap MS	[9]
		*E. australis*	Portugal	Flowering aerial parts	HPLC-DAD and HPLC-ESI-MS	[121]
**11**	Quercetin 3-*O*-rutinoside	*E. multiflora*	Tunisia	Leaves	LC–MS/MS	[117]
**12**	Gossypetin	*E. australis*	Portugal	Flowering aerial parts	HPLC-DAD and HPLC-ESI-MS	[121]
**13**	Luteolin	*E. arborea*	Turkey	Leaves	HPLC-LTQ Orbitrap MS	[9]
		*E. manipuliflora*	Turkey	Aerial parts	LC-MS/MS	[19]
**14**	Isorhamnetin 3-*O*- glucoside	*E. arborea*	Turkey	Leaves	HPLC-LTQ Orbitrap MS	[9]
**15**	Isorhamnetin 3-*O*-α-L-rhamnopyranoside	*E. arborea*	Turkey	Aerial parts	UV, MS, and NMR	[14]
**16**	Tricetin 4′-*O*-α-L-rhamnopyranoside	*E. arborea*	Turkey	Aerial parts	UV, MS, and NMR	[14]
**17**	Kaempferol	*E. arborea*	Turkey	Leaves	HPLC-LTQ Orbitrap MS	[9]
		*E. multiflora*	Tunisia	Aerial parts	HPLC	[85]
		*E. multiflora*	Algeria	Flowered aerial parts	HPLC–DAD–ESI-MS	[16]
		*E. andevalensis*	Spain	Leaves	HPLC	[18]
		*E. australis*	Spain	Leaves	HPLC	[18]
		*E. arborea*	Spain	Leaves	HPLC	[18]
		*E. australis*	Portugal	Leaves and flowers	HPLC	[113]
		*E. australis*	Spain	Leaves, flowers and roots	TLC	[115]
**18**	Kaempferol 3-*O*-glucoside	*E. arborea*	Algeria	Leaves and flowers	HPLC-MS	[24,74,122]
		*E. arborea*	Turkey	Leaves	HPLC-LTQ Orbitrap MS	[9]
		*E. multiflora*	Tunisia	Leaves	LC–MS/MS	[117]
**19**	Kaempferol 3-*O*- rhamnoside	*E. arborea*	Turkey	Leaves	HPLC-LTQ Orbitrap MS	[9]
		*E. australis*	Portugal	Flowering aerial parts	HPLC-DAD and HPLC-ESI-MS	[121]
**20**	Kaempferol 3-*O*-rhamnoside-malonyl-glucoside	*E. multiflora*	Tunisia	Leaves	LC–MS/MS	[117]
**21**	Kaempferol 3-*O*-2G-α-L-rhamnosyl-rutinoside	*E. multiflora*	Tunisia	Leaves	LC–MS/MS	[117]
**22**	Rutin	*E. multiflora*	Morocco	Aerial parts	LC–DAD/ESI–MS	[87]
		*E. multiflora*	Tunisia	Aerial parts	HPLC	[85]
		*E. andevalensis*	Spain	Leaves	HPLC	[18]
		*E. andevalensis*	Spain	Seeds	HPLC	[116]
		*E. australis*	Spain	Leaves	HPLC	[18]
		*E. arborea*	Spain	Leaves	HPLC	[18]
**23**	Apigenin	*E. arborea*	Turkey	Leaves	HPLC-LTQ Orbitrap MS	[9]
		*E. multiflora*	Tunisia	Aerial parts	HPLC	[85]
**24**	Apigenin 7-*O*-glucoside	*E. arborea*	Turkey	Leaves	NMR	[15]
		*E. arborea*	Turkey	Leaves	HPLC-LTQ Orbitrap MS	[9]
		*E. multiflora*	Tunisia	Leaves	LC–MS/MS	[117]
**25**	Apigenin 7-*O*-β-D-(6-*O*-acetyl-glucopyranoside)	*E. arborea*	Turkey	Leaves	NMR	[15]
**26**	Apigenin 7-*O*-D-glucopyranoside	*E. arborea*	Turkey	Leaves	NMR	[15]
**27**	3,5,7,3′,4′,5′-Hexahydroxy-8-methoxyflavone-3-*O*-L-rhamnopyranoside	*E. manipuliflora*	Greece	Aerial parts	NMR	[120]
**28**	3,5,7,3′,4′-Pentahydroxy- 8,5′-dimethoxyflavone-3-*O*-α-L-rhamnopyranoside					
**29**	3,5,7,4′-Tetrahydroxy-8,3′,5′-trimethoxyflavone-3-*O-*α-L-rhamnopyranoside					
**30**	Eriodictyol	*E. arborea*	Turkey	Leaves	HPLC-LTQ Orbitrap MS	[9]
**31**	Taxifolin	*E. arborea*	Turkey	Leaves	HPLC-LTQ Orbitrap MS	[9]
**32**	Taxifolin 3-*O*-rhamnoside	*E. arborea*	Turkey	Leaves	HPLC-LTQ Orbitrap MS	[9]
**33**	Naringenin	*E. manipuliflora*	Turkey	Aerial parts	LC-MS/MS	[19]
		*E. multiflora*	Tunisia	Aerial parts	HPLC	[85]
**34**	Naringin	*E. multiflora*	Algeria	Flowered aerial parts	HPLC–DAD–ESI-MS	[16]
**35**	Aromodedrin	*E. arborea*	Turkey	Leaves	HPLC-LTQ Orbitrap MS	[9]
**36**	Limocitrin	*E. arborea*	Turkey	Leaves	HPLC-LTQ Orbitrap MS	[9]
**37**	Butein	*E. manipuliflora*	Turkey	Aerial parts	LC-MS/MS	[19]
**38**	Phenylethanoid glycosides	*E. manipuliflora*	Turkey	Aerial parts	TLC	[124]

**Table 8:** Catechins profiled in Mediterranean *Erica* species.

No.	Compound	Species	Location	Plant Part(s)	Identification	Reference(s)
**1**	Epigallocatechin	*E. arborea*	Turkey	Leaves	HPLC-LTQ Orbitrap MS	[9]
**2**	Catechin	*E. arborea*	Turkey	Leaves	HPLC-LTQ Orbitrap MS	[9]
		*E. multiflora*	Tunisia	Aerial parts	HPLC	[85]
		*E. andevalensis*	Spain	Leaves	HPLC	[18]
		*E. australis*	Spain	Leaves	HPLC	[18]
		*E. arborea*	Spain	Leaves	HPLC	[18]
		*E. australis*	Portugal	Leaves and flowers	HPLC	[113]
		*E. australis*, *E. arborea*	Spain	Leaves	HPLC	[18]
**3**	Catechin hydrate	*E. manipuliflora*	Turkey	Aerial parts	LC-MS/MS	[19]
**4**	Epicatechin	*E. arborea*	Turkey	Leaves	HPLC-LTQ Orbitrap MS	[9]
		*E. multiflora*	Tunisia	Aerial parts	HPLC	[85]
		*E. andevalensis*	Spain	Leaves	HPLC	[18]
		*E. australis*	Spain	Leaves	HPLC	[18]
		*E. arborea*	Spain	Leaves	HPLC	[18]
		*E. arborea*	Algeria	Leaves and flowers	HPLC-MS	[24,74]
		*E. australis*	Portugal	Leaves and flowers	HPLC	[113]
		*E. andevalensis*	Spain	Seeds	HPLC	[116]

**Table 9:** Anthocyanidins profiled in *E. australis*.

No.	Compound	Species	Location	Plant Parts	Identification	Reference
**1**	Delphinidin 3-5-*O*-diglucoside	*E. australis*	Portugal	Leaves and flowers	HPLC	[113]
**2**	Delphinidin 3-*O*-glucoside					
**3**	Cyanidin 3,5-*O*-diglucoside					
**4**	Cyanidin 3-*O*-glucoside					
**5**	Pelargonidin 3,5-*O*-diglucoside					
**6**	Pelargonidin 3-*O*-glucoside					
